# A Grassroots Remote Sensing Toolkit Using Live Coding, Smartphones, Kites and Lightweight Drones

**DOI:** 10.1371/journal.pone.0151564

**Published:** 2016-05-04

**Authors:** K. Anderson, D. Griffiths, L. DeBell, S. Hancock, J. P. Duffy, J. D. Shutler, W. J. Reinhardt, A. Griffiths

**Affiliations:** 1 Environment and Sustainability Institute, University of Exeter, Penryn Campus, Cornwall, United Kingdom; 2 FoAM Kernow, Jubilee Warehouse, Penryn, Cornwall, United Kingdom; 3 Centre for Geography, Environment and Society, University of Exeter, Penryn Campus, Cornwall, United Kingdom; University of Maryland at College Park, UNITED STATES

## Abstract

This manuscript describes the development of an android-based smartphone application for capturing aerial photographs and spatial metadata automatically, for use in grassroots mapping applications. The aim of the project was to exploit the plethora of on-board sensors within modern smartphones (accelerometer, GPS, compass, camera) to generate ready-to-use spatial data from lightweight aerial platforms such as drones or kites. A visual coding ‘scheme blocks’ framework was used to build the application (‘app’), so that users could customise their own data capture tools in the field. The paper reports on the coding framework, then shows the results of test flights from kites and lightweight drones and finally shows how open-source geospatial toolkits were used to generate geographical information system (GIS)-ready GeoTIFF images from the metadata stored by the app. Two Android smartphones were used in testing–a high specification OnePlus One handset and a lower cost Acer Liquid Z3 handset, to test the operational limits of the app on phones with different sensor sets. We demonstrate that best results were obtained when the phone was attached to a stable single line kite or to a gliding drone. Results show that engine or motor vibrations from powered aircraft required dampening to ensure capture of high quality images. We demonstrate how the products generated from the open-source processing workflow are easily used in GIS. The app can be downloaded freely from the Google store by searching for ‘UAV toolkit’ (UAV toolkit 2016), and used wherever an Android smartphone and aerial platform are available to deliver rapid spatial data (e.g. in supporting decision-making in humanitarian disaster-relief zones, in teaching or for grassroots remote sensing and democratic mapping).

## Introduction

Smartphones are powerful research tools for collecting scientific data because they are equipped with a broad suite of sensors (e.g. cameras, microphones, light sensors, accelerometer, compass, gyroscope, and GPS) and on-board microcomputers and are globally ubiquitous. ‘Mobile phone sensing’ [1] is emerging as a new field of research and a growing number of scientists are developing ways of exploiting the technological capabilities of smartphones. Examples include social science investigations [2], human population mapping for epidemiology [3,4], ecological data capture [5], atmospheric monitoring [6] and clinical studies [7,8]. Smartphones are typically equipped with a broad suite of sensors, designed to service the information requirements of users and multinational developers alike–they are location-aware, and applications downloaded by users can transmit information back to providers. This is a capability that can be exploited through the programmable nature of smartphones—sensors developed for providing location-based services can now be hacked using readily available computing resources that offer a “low barrier of entry for third-party programmers” [1].

A somewhat untapped opportunity lies in converting the smartphone into an all-inclusive or self-contained remote sensing device that not only captures images, but also records the metadata from various phone sensors coincidently (e.g. GPS, and sensor attitude data). Remote sensing is the science of acquiring spatial, spectral or temporal information about objects without making physical contact with the object. Typically remote sensing data comprise images acquired from sensors on platforms such as piloted aircraft or satellites but a new self-service, and to some extent, ‘grassroots’ (participatory and distributed) remote sensing revolution is underway making use of drones and kites as platforms for proximal observations of environmental phenomena [9–11]. These platforms cannot carry the heavy payloads used on satellites or aircraft, but they offer a more flexible means of timely and responsive survey, and their low flying capability means that high spatial resolution data can be captured easily. For example in the aftermath of a humanitarian disaster the ability to rapidly and cheaply survey damage and search for survivors is more readily achieved from a lightweight and portable platform like a drone or a kite [12] than from a piloted aircraft or satellite. The resolution of data from low-flying platforms such as drones and kites means that the data are more useful and easily interpreted in such settings. Additionally smartphones provide a means for participatory map making in places where global datasets (e.g. Google Earth) lack spatial fidelity, and where community tools (e.g. OpenStreetMap) provide platforms for sharing open source spatial information amongst individuals [13,14]. Indeed, movements such as Humanitarian Street Map (https://hotosm.org/) could benefit greatly from the low-cost digital mapping opportunity offered a kite or lightweight drone equipped with a ready-to-use smartphone for capturing aerial data.

The *status quo* for drone- and kite-based aerial mapping is to use either camera systems with wired triggers (e.g. where a camera is triggered to take a photograph by an on-board autopilot), cameras fitted with intervalometers, or cameras hacked to give improved autonomous functionality (e.g. using tools such as the Canon Hack Development Kit). A key challenge is enabling the camera to capture images without the need for human intervention once airborne. Data processing then uses complex software (e.g. ‘structure-from-motion’ software) to convert the resulting aerial photography data into ready-to-use orthorectified maps and three-dimensional point clouds, where required [15–18]. Whilst these approaches generate high quality scientific products, for many basic mapping applications the workflow is too complex (e.g. wiring the camera to an autopilot trigger is non-trivial, and the post-processing stage requires high performance software and computing), and the detail in and complexity of the resulting products exceeds what is really needed. For many applications basic aerial photography data are sufficient to enable identification of landscape features and to map environmental patterns [19]. This paper is concerned with developing an ‘appropriate technology’ [20] toolkit for basic autonomous image capture using a mobile phone. We asked: can a basic smartphone, with its plethora of on-board sensors (accelerometer, GPS, compass, camera) be used to generate ready-to-use spatial data from lightweight aerial platforms such as drones or kites? Smartphones contain everything necessary to allow geo-tagged images to be delivered, being equipped with high resolution digital cameras, on-board computer processors, GPS receivers, and typically accelerometers and a compass. There are a variety of useful opportunities in combining data from these various sensors and so, we sought to develop an application that converted a smartphone into an autonomously triggered drone- or kite-ready map-making device.

This paper describes the development, testing and evaluation of a bespoke smartphone app for automatically collecting and generating geotagged aerial images, and suitable for deployment on any lightweight drone or kite aerial photography (KAP) rig. The manuscript details the technical approach, the results of field tests on kites and lightweight drones and discusses the challenges with deployment and data processing. The code and app developed are open-source and freely available for use.

## Methods and Materials

The app was designed to be implemented on an Android smartphone handset. Android provides the most flexible platform, allowing the app to be developed as an open-source toolkit. At the time of development, the global distribution of Android smartphones greatly exceeded that of other operating systems (in the first quarter of 2015 the global market share of Android was reported to be 78% by the International Data Corporation [21]), therefore offering the most ubiquitous platform for global uptake.

A limited number of similar smartphone apps for remote sensing exist. Those that do exist are primarily for citizen science applications where ground-based validation data are needed to help improve the quality and validity of products such as global land cover maps. Two such apps are ‘Field Photo’ [22] and ‘GeoWiki Pictures’ [23]. Both offer a route for users to capture geo-tagged images of landscape features, allowing the organisations who administer the apps to use these data for broader purposes. In both cases, the user is required to trigger the camera on the handset, and the app then records various metadata describing the conditions and location of the photograph. In ‘Field Photo’ the app simply logs the GPS location whilst ‘GeoWiki Pictures’ also captures metadata describing the compass angle and direction of tilt. Neither app allows the user to programme the conditions under which the camera is triggered or to allow the device to trigger automatically. Equally, both of these apps are static, and developments and changes can only be implemented by the developers. It was these two functions that were critical to our application:

1. The need for a user to press a button to trigger data capture is not feasible when the device is airborne, so it was essential that our app would allow the camera and sensors on the smartphone to function autonomously after set-up
2. A ‘fixed’ app that prevents the user from making changes to the way images are captured can be restrictive in operational settings, so we wanted to make this flexible and programmable by the end-user in an accessible way.

## Critical design steps

In designing the app it was first necessary to identify the critical steps to turn a standard Android smartphone handset into a remote sensing device. A key focus of the design was to trigger the camera to capture photographs automatically, whilst simultaneously recording metadata describing other conditions (position, direction, angle of acquisition, time). Several steps were followed in the design of the app for this purpose. The technical specification of the app dictated that it had to be able to:

1. Identify and capture information about the full suite of sensors on the smartphone (which varies between handset models).
2. Capture information from the smartphone describing the camera model (field of view, pixel resolution, shutter speed).
3. Control the camera so that it could be triggered to acquire images given a set of pre-defined capture parameters (e.g. a timed trigger or a positional trigger).
4. Simultaneously record data from the phone’s GPS to geolocate and timestamp each image, and use the accelerometer and compass to record the attitude (pitch, roll and heading) of the phone such as would be achieved in a typical ‘aircraft attitude’ data stream on board a remote sensing survey aircraft, or by a drone autopilot.
5. Store metadata as linked records to each individual photographic image.

A second phase of development focused on developing a user-configurable app. This was considered essential because a fixed format application that could only generate time-triggered image capture, or GPS-triggered image capture would not necessarily suit all potential users. An intention of this project was to work more closely on a flexible app design that allowed users to change the conditions under which the camera trigger was activated–allowing, for example, attitude-based quality control (e.g. only capture an image if the camera is level and pointing downwards). To enable this flexible functionality, a visual coding methodology was followed.

A third phase of development was to experiment with an open-source approach for post-processing the captured images into Geographical Information Systems (GIS)-ready geo-TIFFs, after the images had been downloaded from the smartphone. This approach required new code to convert the image jpeg into GIS-format data.

### Visual coding methodology

The conventional app design process assumes that the purpose of the system being designed is fixed, and a programmer’s job is to provide features that solve existing problems for a client. In the case of experimental research, and in this project, these relationships are not so clearly defined and this led us to pursue a more flexible approach to the app design, specifically drawing on research in visual and live coding. Visual coding is a way of enabling simple programming by manipulating program elements graphically rather than by through text definition. Visual coding allows for end-user development (EUD; a more flexible approach to software production by people with expertise in other domains, and those working towards goals supported by computation [24]) of apps through live coding (where coding is designed and implemented on the fly). Live coding is widely used within the arts, for example, in music performance [25,26] and is increasingly used as a tool for teaching introductory programming [27]. As a programming tool itself, live coding can result in many new, flexible approaches to programming. Live coding was trialled here to allow for EUD within the app. We developed an approach that allowed users to program the app using the phone’s touch screen, using a system based on ‘Scheme Bricks’ [28] which utilises drag and drop positioning of logical blocks to construct expressions which are evaluated in flight by a tinyscheme [29] interpreter. Blocks are picked using Android's ‘long press’ function, and haptic feedback was provided by vibration to indicate successful selection.

The nature and quantity of sensors in mobile devices are changing rapidly and the suite of sensors available varies from one handset to another. For this reason, we did not wish to stipulate that a specific sensor set should be required to allow the app to work, but the minimum requirement was GPS and a camera. The addition of numerous combinations of other sensors was anticipated to enhance the remote sensing approach, so flexibility in calling on a variety of sensors was an advantage that was only possible through use of an EUD approach. In many survey settings, it is also helpful to be able to change the behaviour of the software to perform specialist functions. Such flexibility in the app design is particularly important in a research context where researchers might also have experience of scripting and programming and wish to make their own changes. Finally, with airborne mapping there are sometimes local conditions (e.g. variable wind, changing light conditions) that require different camera triggers or imaging behaviour. The ability to make adjustments to the program behaviour in the field without requiring a full development toolchain (e.g. laptops and cables) was therefore highly attractive.

The app was designed with a completely open EUD capability whilst also offering three example programs for the most commonly used modes of image capture: a simple timer to trigger photo capture every three seconds whilst simultaneously recording sensor data describing the orientation and GPS position of the images; and two further programs that calculated the overlap of photos using different methods, driven by the on-board GPS to mitigate against the app collecting too many photographs. For EUD four kinds of programming block were available:

- **Triggers:** these are time or space based blocks that go on the top level of a program to trigger subsequent actions. They trigger based on a simple timer (when-timer), distance covered using GPS (when-moved-metres) or distance from all previous trigger locations (when-in-new-location).
- **Actions:** used to display data for debugging, for feedback–sound/vibrate, or to record data to the database.
- **Sensors:** the app lists all types of sensors on-board the smartphone, as exposed by Android's OS (for example, these may include sensors such as: accelerometer, temperature, gravity, gyroscope, light, linear acceleration, magnetic field, orientation, pressure, proximity, relative humidity, rotation vector, significant motion, camera, and GPS coordinates).
- **Mathematical expressions:** are provided for calculations to be carried out in flight, for example the coverage of a photograph based on the camera angle and altitude.

### Tests on android smartphone platforms

Two smartphones were used to test the app–a low-cost Android handset (Acer Liquid Z3) and a more advanced Android handset (OnePlus One A0001). Both were chosen because they offered the necessary basic sensors for aerial photographic survey–a GPS and a camera. The two smartphones were also chosen because they offered different capabilities in terms of other on-board sensors, with the OnePlus One A0001 model offering a richer set of on-board sensors for metadata collection. Table 1 shows the basic specifications of each smartphone.

**Table 1:** Specifications of the two test smartphones used in this study. **All information taken from** http://www.gsmchoice.co.uk/ [accessed 4 August 2015].

Functionality	Acer Liquid Z3	OnePlus One A0001
Dimensions (w,h,d)	109.9 x 60.0 x 10.4 mm	152.9 x 75.9 x 8.9 mm
Weight	120 g	162 g
Main camera, matrix	3 megapixels	13 megapixels
Main camera, resolution	2048x1536 pixels	4128x3096 pixels
Video capability?	Yes	Yes
Internal and RAM memory	4 Gb; 512 Mb	16 Gb; 3Gb
Operating system	Android 4.2 jellybean	Android 4.4 kitkat
Battery	Li-Ion 1500 mAh	LiPo 3100 mAh
GPS	Yes, basic A-GPS only	Yes, A-GPS with additional capability for GLONASS and Beidou constellations, plus Near Field Communication (NFC).
Processor	MediaTek MT6572M, processor clock: 1.00 GHz, number of cores: 2, GPU: ARM Mali-400 MP1 @500 MHz	Qualcomm Snapdragon 801 8974AC, Processor clock: 2.50 GHz, Number of cores: 4, GPU: Adreno 330 @578 MHz

Automatic image capture is considered a privacy problem in normal smartphone use and thus is contrary to usual Android design guidelines. A camera timer mode had to be coded to over-ride this restriction. In addition we needed to take steps to prevent accidental button presses or screen activity during flight or attachment to the vehicle in use. The app therefore included a 'camera lock' feature that made the app full screen to prevent operating system (OS) navigation buttons being pressed whilst in use. The app could then be stopped by using a long-press on the power button to cease data capture. Both the key-guard screen and rotation layout changing was disabled by the app, because this was found to interfere with the camera functionality in some cases. The behaviour of these actions varies from phone to phone based on the specific version and manufacturer but these steps worked well with the test phones we used (Table 1) and ensured robust and continual functioning of the app in demanding field situations.

### Flight tests and platforms

We tested deployment on a range of lightweight (sub-7kg take-off-weight) drones and two different kites—details of the systems used are provided in Table 2. It is important to note that we were focused on testing the app’s performance in very basic settings, acknowledging that the idea behind the research was to make and test the utility of the procedure for grassroots spatial mapping. For this reason, we tried to avoid using bespoke holders or specialised mounts to attach the smartphone to the airborne platform, instead focusing on using attachments or fittings already on the drones, or easily made for kites. This allowed us to test the performance of the technology in settings that were most realistic given the intended audience.

**Table 2:** Airborne platforms used for testing the app and the various sites where flight tests were performed.

Platform	Make / model / type	Cost	Fixing	Field testing sites
Multirotor battery-powered drone	3D Robotics Y6 hexacopter	£600	Attached to the under-carriage beneath the LiPO battery, using velcro straps.	Mixed coastal grassland site adjacent to a steep cliff.
Multirotor battery-powered drone	Quanum nova quadcopter	£300	Attached under the body of the aircraft using hardware tape and cable/zip ties.	Mixed grassland with sparse woodland.
Fixed wing radio controlled, glo-fuel powered aircraft	Flair Cub aircraft (balsa wood frame)	£300	Secured with tape on the underside of the aircraft’s nose. Two flight modes–powered and in a controlled glide descent.	Mixed coastal grassland site adjacent to a steep cliff.
Fixed wing battery-powered glider aircraft	X-UAV SKUA (polystyrene frame)	£250	Secured to the end of the wing using hardware tape.	Mixed coastal grassland site adjacent to a steep cliff.
Stunt kite	Flexifoil	£100	A handmade rig consisting of a wooden strip hung between the two kite lines. The phone was secured to the wooden strip using hardware tape.	Beach with rocky outcrops and tidal pools.
KAP single line kite	HQ KAP foil 1.6m	£80	3D printed holder with four corner attachments, looped onto the single line using alpine butterfly knots. The phone was attached to the holder using hardware tape and cable/zip ties.	Two sites were used, first an old quarry area with small ponds, aggregate piles and sand-dune vegetation, and secondly a beach with rocky outcrops and tidal pools.

**Fig 1:**
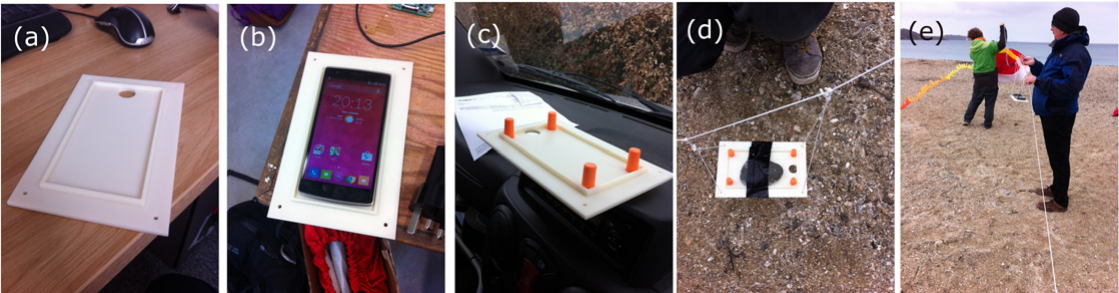
A 3D printed holder for the OnePlus One handset for suspension from a single line KAP kite. (a) the bare 3D printed holder (b) with the smartphone positioned (c) with superglued earplug dampeners to allow compression to fix the phone in place (d) with a stone taped to the jig to test the kite’s fly-ability with a payload, and fixed using alpine butterfly knots to the kite’s line prior to first deployment of the phone and (e) with the phone fixed using cable/zip ties prior to the first KAP test flight on a beach showing two of the authors preparing to launch the kite.

**Fig 2:**
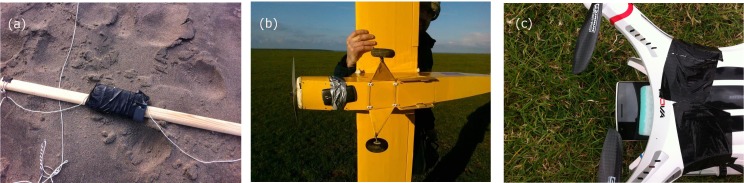
Examples of basic fitting methods to hold the mobile phone handset in place on airborne platforms. Shown are (a) a wooden bar suspended from two strings on the flexifoil stunt kite with the OnePlus One phone attached using hardware tape (b) the Acer Liquid Z3 taped to the underside of the balsawood Flair Cub aircraft with hardware tape and (c) the OnePlus One phone attached using hardware tape to the underside of the Quanum Nova lightweight quadcopter with a sponge to reduce image blurring caused by airframe vibration.

For the KAP platform (see Table 2), we developed a customised part to hold the OnePlus One handset. A plate was designed and printed using a 3D printer to provide a bespoke holder for the OnePlus One phone. A hole was left for the rear camera and four holes were made in each corner for hanging the rig from the kite using alpine butterfly knots. Fig 1 shows the detail of this 3D printed part. For the other platforms, rudimentary fixings were used to ensure safe fitting of the handset to the drone or kite, whilst also relying on minimal specialist equipment. Fig 2 shows three examples of how the OnePlus One phone handset was fixed to various platforms.

### Development of a post-processing methodology for GeoTIFFs

The final stage in the process was convert the images captured by the phone into GIS-ready GeoTIFFs. This part of the process sought to determine the extent to which the metadata from the phone’s other sensors could be used to rotate and then fit the photographs to a gridded map projection and is a completely novel and bespoke approach for which existing software is not available. This process was developed and tested using a simple python script alongside freely available and open-source tools from the Geospatial Data Abstraction Library (GDAL [30]). Three steps were followed:

1. The sensor metadata captured simultaneously by the app and stored in the on-phone database were extracted and stored as a SQLite database. The database was used to match metadata records to specific photographs.
2. The metadata in the SQLite database were then assimilated into GDAL and converted using gdal_translate [30] to generate a set of ground control points describing the geographic location of each corner of each image. We experimented with various sensor combinations to determine which produced the best results.
3. The result of 2 was then fed into the ‘gdalwarp’ utility tool [30], once to process the pixel data and a second pass to convert the coordinates to EPSG:3857 (a geographic projection commonly used for web-mapping applications) so that the orientation and position of the images could be checked against independent aerial photography datasets of the same site.

### Open source reporting and development changes

In the spirit of grassroots and open-source movements, the app is free for any Android user to download. The process of source control and a full commit log for the entire process describing the development of the app is available and freely accessible through a Github repository and is updated each time changes are made to the app [31].

## Results

### Visual coding results

**Fig 3:**
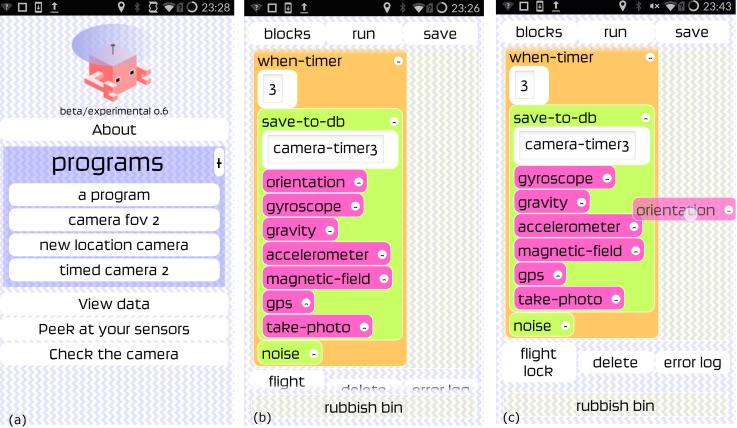
Screenshots from the app showing (a) the opening screen (b) the coding scheme bricks and (c) an example of live coding where a scheme brick is in the process of being moved within the program.

**Fig 4:**
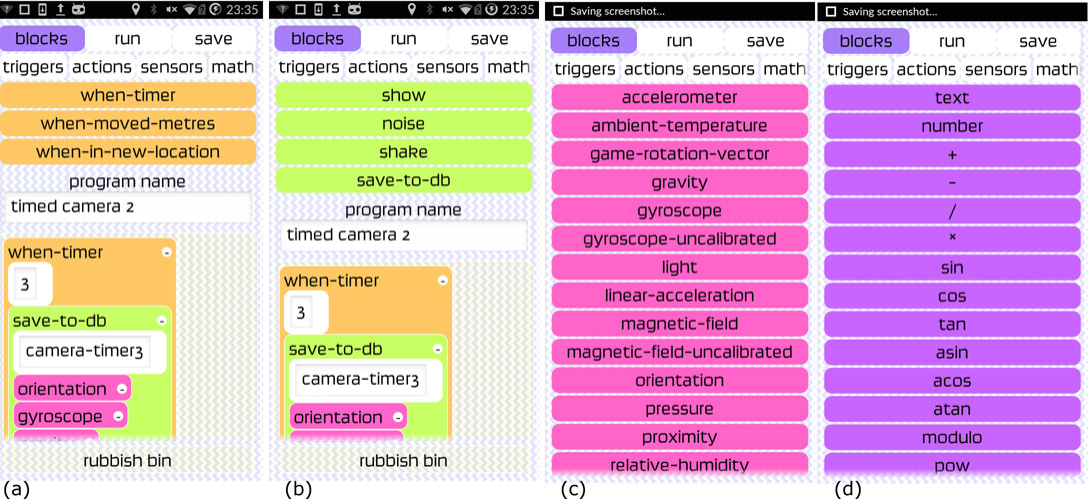
Examples of the coding blocks that could be chosen on the OnePlus One handset where (a) is the triggers, (b) shows actions that the phone can perform when a trigger is set (c) demonstrates sensor selection and (d) shows mathematical operators.

**Fig 5:**
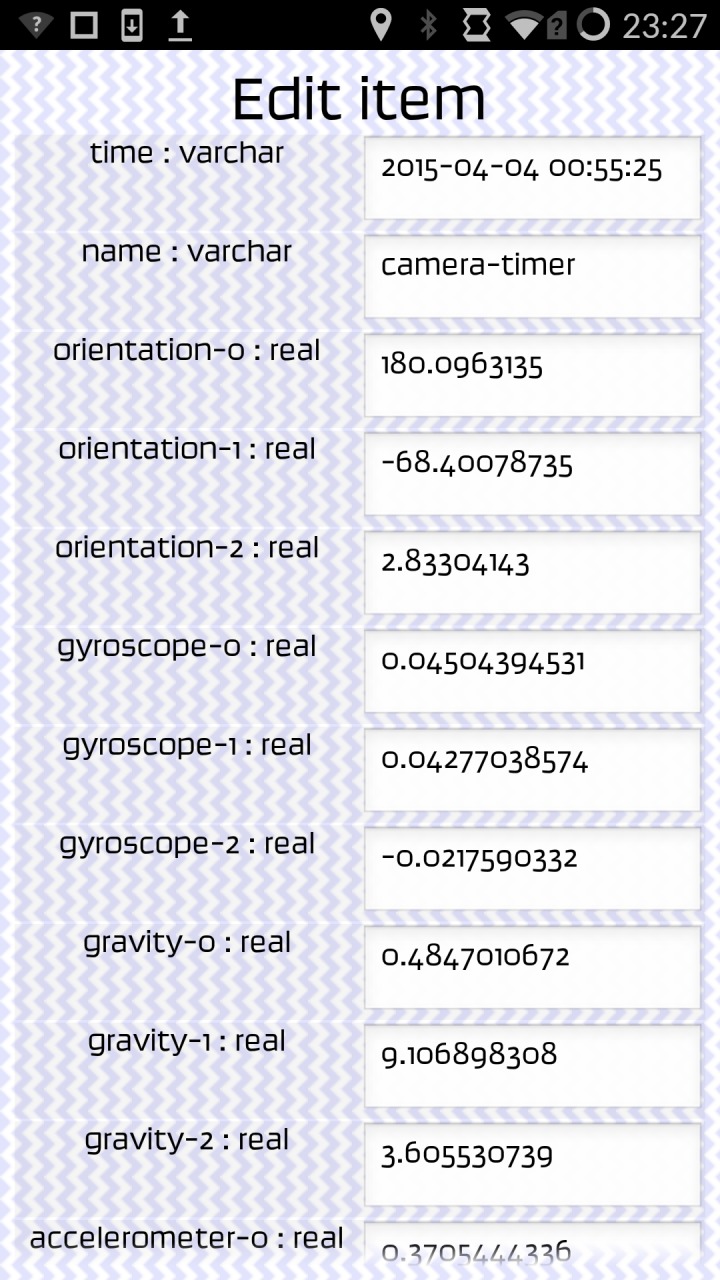
Example metadata shown on the screen of the OnePlus One phone.

Fig 3 shows screenshots from version 0.6 of the app. Fig 3(A) shows the opening screen of the app whilst Fig 3(B) shows the coding blocks for a 3 second camera trigger where various other sensors are also triggered and a ‘ping’ noise is activated per acquisition. Fig 3(C) shows an example of live coding where the orientation sensor has been ‘picked’ by the user to move it around within the coding framework. The buttons shown in Fig 3(C) at the bottom of the app screenshot show the ‘flight lock’ button which can be activated once the program is running (bottom right button)–this was designed to deactivate all other activity on the phone so that the app could run without being interfered by other processes. The rubbish bin icon/block at the bottom of Fig 3(B) and 3(C) is where unwanted blocks can be placed during live coding. Fig 4 shows the different block types available for live coding the app. Fig 5 shows the metadata recorded by the app for a single test acquisition, displayed when the ‘view data’ (See Fig 3(A)) button was pressed within the app. This gives the user of the app an immediate *in situ* view of the data stream from the phone sensors.

### Results from flight tests

#### Results from powered lightweight drones

**Fig 6:**
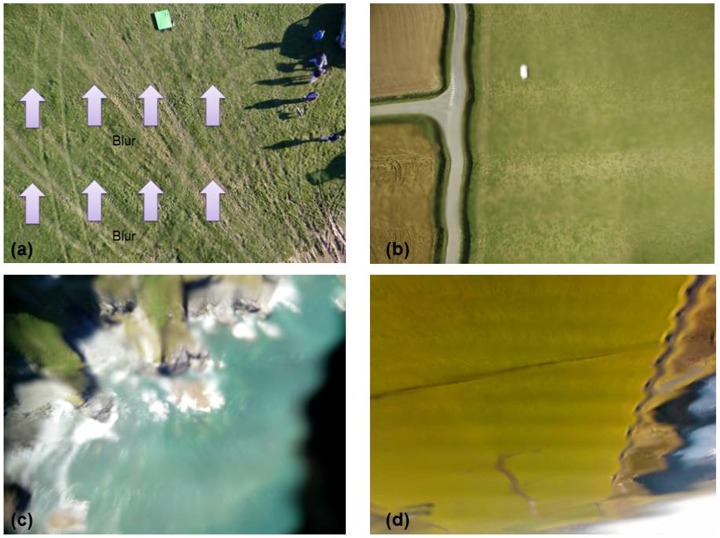
Vibration issues caused image blurring when the phone handset was not vibration dampened. (a) grassland image captured by the Acer Liquid Z3 handset flown on 3D Robotics Y6 airframe (b) a road and fields captured from the OnePlus One handset on SKUA airframe; (c) coastal cliffs from the OnePlus One handset on a petrol powered Flair Cub airframe; (d) agricultural area captured from the Acer Liquid Z3 on the Flair Cub airframe.

Flight tests from powered lightweight drone platforms generated a range of results which were used to evaluate the performance of the app. The main issue with deployment on powered drones (both multi-rotor and fixed wing aircraft) was vibration in flight. Due to the fact that we used simple, non-vibration dampened fixings for the phones there was always some in-flight vibration which transferred to the devices. This gave rise to a range of image quality issues, as shown in Fig 6. In Fig 6(A) we show an annotated image of a grassland test site collected using the Acer Liquid Z3 handset attached to the 3D Robotics Y6 airframe. The arrows indicate where image blurring occurred in stripes and caused subtle distortions in the image. These distortion lines were present in most of the data collected from powered drones to a greater or lesser extent, and were always aligned with the camera pixels rather than orientated in the direction of platform motion. Fig 6(B) was collected from the OnePlus One phone from the fixed wing SKUA aircraft. It shows a road adjacent to some agricultural fields, where similar image blurring is obvious relative to the linear feature of the road. The blurring and geometric distortion was oriented across the image from left to right as is indicated by the road junction where the left hand road is less severely distorted than the road running from top-to-bottom of the image. Fig 6(C) was collected by the OnePlus One phone taped to the underside of the Flair Cub aircraft as it flew over some coastal cliffs. The black area to the bottom right of the image was the tape (used for fixing the phone to the aircraft)–and it is clear that the straight edge of the tape has been captured as being non-straight due to vibration, causing image distortion. Similarly, the geometric properties of the cliffs below are poorly captured due to vibrations from the petrol motor. Fig 6(D) was captured by the Acer Liquid Z3 from the same Flair Cub platform over agricultural fields and the same geometric distortion is clearly visible.

Initially, the app was designed to vibrate at the point of image collection as a haptic indicator that the app was triggering. The early results (Fig 6(A)) which showed blurring in the images led us to change the coding of the app so that a ‘ping’ sound alert indicated successful image capture instead of a haptic vibration cue. In subsequent flights, with this ‘ping’ modification running, we found that this change in functionality had no noticeable effect on image quality (Fig 6(B) to 6(D)), indicating that the vibration of the platform was a more significant issue. We suggest that these image distortions were due to the interlaced sampling of the camera sensor imaging array (in both cases, these were Complementary Metal Oxide Semiconductor (CMOS) sensors [32]). Furthermore these distortions could also have been an artefact of the CMOS image stabilization algorithm not being capable of overcoming high levels of vibration from powered drones. The results suggest that vibration dampening would be an important feature governing successful deployment of the app on lightweight drone platforms in operational situations.

**Fig 7:**
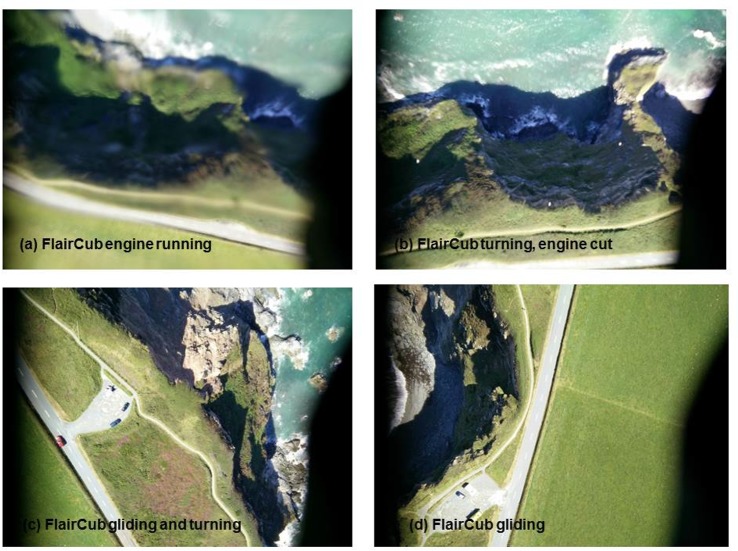
Results of an in-flight experiment to test the impact of engine vibration on image quality using the OnePlus One phone and the Flair Cub platform when (a) the engine was running, (b) the engine had just been turned off and (c), (d) when the aircraft was gliding and vibration distortions were minimised. The black ‘edge’ shown bottom right was a piece of tape used to fix the phone in place on the aircraft, and was also used as a constant visual guide for the level of image distortion encountered in the different scenarios. The edge gets sharper as the engine is turned off and the aircraft is switched to gliding mode.

**Fig 8:**
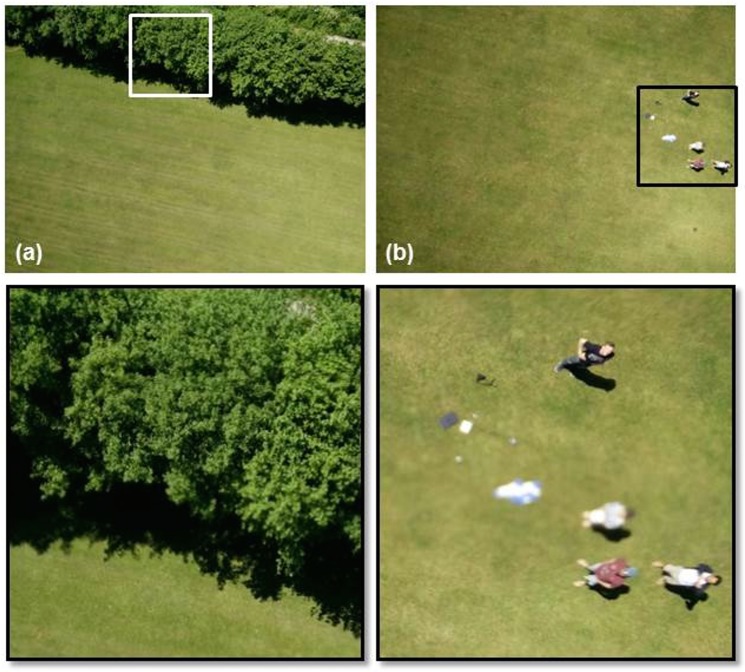
Example images over a mixed grassland/woodland site collected by the OnePlus One phone fixed to the Quanum Nova quadcopter where vibration dampening was achieved using a kitchen sponge between the phone handset and the aircraft (see Fig 2(C)). (a) is an area of grass bordered by trees and (b) shows the flight crew on the ground. Below (a) and (b) are two areas of focus, showing (a) good quality data with minimal blurring over a patch of trees and (b) evidence of some blurring in an image collected over the flight team (all co-authors of this paper).

To test the hypothesis that the blurring and image quality issues were caused by engine vibrations we used the Flair Cub platform to capture some test images during a flight over a coastal grassland site with some nearby cliffs (the same site shown in Fig 6(C)), both with the engine running and then in a controlled glide. A time series of four images captured during that test flight are shown in Fig 7, beginning top left (a) with the engine running, showing the effect of engine vibration and then, through (b) where the engine had just been cut, through (c) and (d) when the aircraft had just turned and was beginning a controlled glide to descend for landing. Fig 7 evidently shows much higher image quality captured after the engine was cut ((c) and (d)). The linear features of the road and the detail in the cliff structures are much more clearly discernible and the images have greater geometric clarity without the distortions to linear features previously evident in Fig 6(B), for example. Using the straight edge of the piece of black tape that partially covered the lower right hand part of the lens as a secondary guide it is possible to see from Fig 7 that the photographs captured whilst gliding (7(c) and (d)) suffered less distortion than those captured when the engine was running (7(a)). Fig 8 provides two photographs (with zoom areas shown beneath) collected by the OnePlus One phone when flown on the Quanum nova quadcopter. Here, we sought a low-cost option to dampen vibrations, placing a kitchen sponge (as shown in Fig 2(C)) between the phone and the aircraft underside to absorb vibrations. Whilst some image quality issues remained (blur is evident in the zoomed area in (b)), the overall mapping capability of the phone on this low cost platform appeared good and certainly typical results were improved (e.g. zoomed area in (a)) over the blurry examples shown in Figs 6 and 7.

#### Results from kite platforms

**Fig 9:**
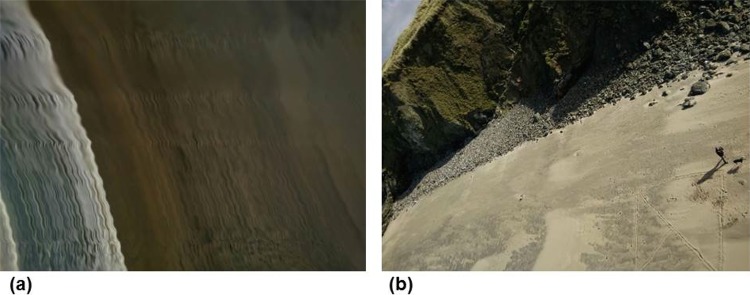
Results from a Flexifoil kite where the OnePlus One phone was attached to the wooden jig shown in Fig 2(A). (a) Shows the impact of high winds and vibration over a sand and sea scene, and (b) shows a clearer image captured from an off-nadir geometry of one of the authors and a cross-hair target carved into the sand.

Results from tests on kite platforms were also informative and helped to define the operational limits of the app. For all kite tests, the OnePlus One handset was used. Fig 9 shows early results from the Flexifoil stunt kite (fixing shown in Fig 2(A)). In Fig 9(A) an image collected over a sandy beach (with the sea on the left of the image and the beach on the right). Here, the impact of kite vibrations was very clear–the conditions on the day of this flight were windy, with gusts up to 20 miles per hour, and coupled with the wooden jig which restricted pilot control over the kite, we found that the platform was not ideal for aerial photography, and that the app was unable to cope well with the highly variable conditions. Despite this, some useable images were captured from the Flexifoil platform–Fig 9(B) shows an off-nadir image with a cross-hair target carved into the sand where the image geometry and basic features of this coastal setting were well photographed. It is important to state that the image in Fig 9(B) was the exception, rather than the norm of this particular survey.

**Fig 10:**
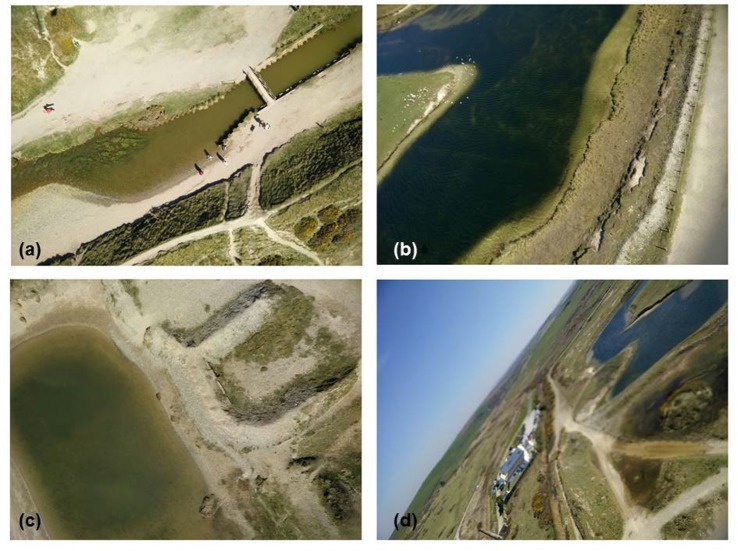
Successful images captured by the app during deployment at a disused quarry area with small ponds, aggregate piles and sand-dune vegetation. (a) to (c) show good quality nadir-images captured by the app whilst (d) shows an off-nadir image of the site captured when the phone holder was moved in a gust of wind.

**Fig 11:**
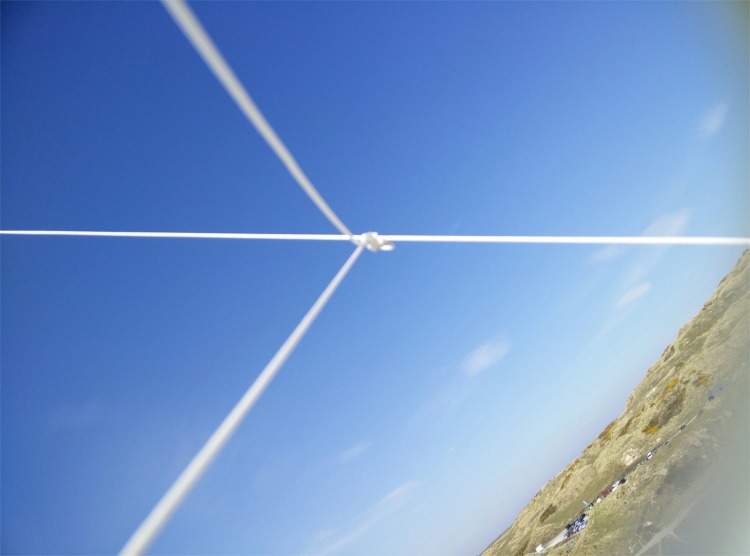
Example ‘kite line’ image captured with the OnePlus One phone from the KAP rig when the phone holder became inverted during a gust of wind.

Improved results were captured from a single line KAP kite–example images are shown in Fig 10 from a coastal, unused quarry area with small ponds, aggregate piles and sand-dune vegetation. Here, the phone was suspended using the 3D printed mount (Fig 1) from the single line kite, and the kite flown in approximately 20 mph onshore winds at an estimated height of 30 m. Weather conditions on this date were optimal for photography with clean blue skies, and images were captured close to midday when the sun was at peak zenith. The images shown in Fig 10(A) to 10(C) show no evidence of the geometric or vibrational distortions found with other platforms. Fig 10(A) shows a small stream (approximately 3 m wide at the bridge) passing through a sand-dune area; Fig 10(B) shows a pond within the quarry site with birds obvious on the island at centre left and a pathway running along the right hand edge of the image; and Fig 10(C) shows a small shallow pond with neighbouring sand dune vegetation. In all three cases, the images captured were sharp and of a very high quality geometrically. The image shown in Fig 10(D) was an off-nadir image captured when the kite was caught in a short-lived gust of wind, which caused the holder to spin around–it is geometrically poor but offers a useful overview of the site’s locality. During two kite flights of this area over a 2 hour period, we collected 585 photographs of the site. Of those, 124 were deemed unusable due to the presence of kite string within the images (Fig 11 provides an example) caused by the phone becoming inverted during strong gusts of wind. On subsequent flights, we painted a black cross on the underside of the phone holder so that it was obvious to a ground observer if the holder had become inverted. Action could then more easily be taken to try to flick the holder back the right way, or bring the kite down so that a re-launch could be achieved with the phone in the right geometry.

**Fig 12:**
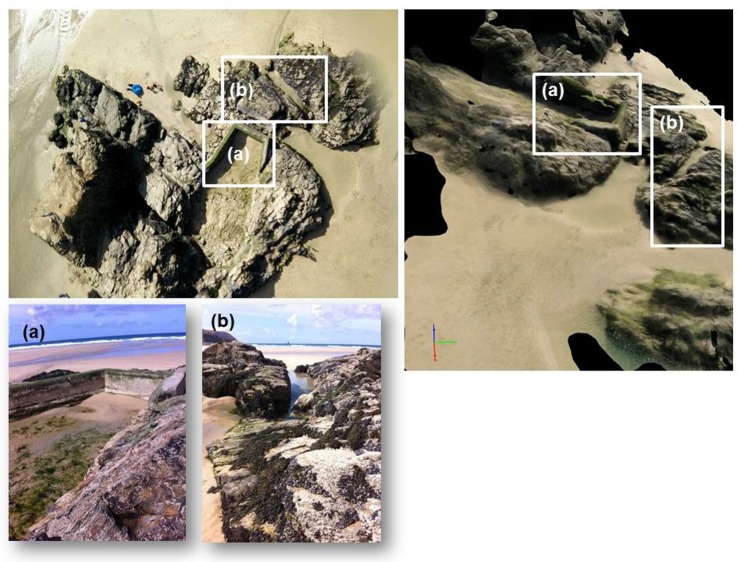
Result of stitching 19 images collected by the OnePlus One phone attached to the KAP rig using Pix4D software into a basic SfM product. Features (a) and (b) are labelled in one of the original photographs (top left), in the resulting SfM model (top right) and shown in field photographs (bottom of figure) for comparison.

Finally a series of test flights with the same KAP rig over a rocky coastal outcrop were undertaken, resulting in the data shown in Fig 12. A total of 19 crisp images were captured over the main rocky outcrop shown in the top left image in Fig 12 (example KAP photograph captured by the app) in gusty winds up to 25 mph from an approximate height of 30 m. These images were tested within Pix4D (commercial software for image stitching and for ‘structure from motion’ (SfM) model generation [33–35]) to determine whether they could be used to produce a useful spatial model of this coastal feature. The resultant 3D model (taking less than 20 minutes processing time on a standard windows 64bit desktop PC) is shown visually on the right of Fig 12. Areas labelled (a) and (b) are shown as photographed from the ground, (a) is a tidal swimming pool with a concrete wall and (b) is a natural crack in the rock platform with a distinctive structure. Whilst we have not quantitatively evaluated the quality of the SfM product, it is clear from visual assessment of the constructed model that these features are reasonably well represented in the resultant model. This is the first time that SfM has been demonstrated to work with images captured from a mobile phone using an autonomously triggered app, and flown on a KAP rig.

#### Results of GeoTIFF processing using phone-gathered metadata

Example metadata as stored in the SQLite database and collected by each of the two test handsets are shown in Table 3 for two separate acquisitions. Note that the sensor set available for the Acer handset was much reduced compared to the higher specification OnePlus One handset.

**Table 3:** Example metadata records from the two handsets tested in the study.

Metadata label	OnePlus One	Acer Liquid Z3
time	2015-04-08 20:45:25	2015-03-30 14:07:00
name	camera-timer2	camera-timer
orientation-0	318.4345093	82.65625
orientation-1	-5.552710533	-1.453125
orientation-2	-1.676241875	-0.84375
gyroscope-0	0.088394165	no data
gyroscope-1	1.642929077	no data
gyroscope-2	1.160018921	no data
gravity-0	-0.286861807	no data
gravity-1	0.94849962	no data
gravity-2	9.756456375	no data
accelerometer-0	1.02947998	-0.149160877
accelerometer-1	4.882263184	0.257641524
accelerometer-2	10.92797852	10.11581993
magnetic-field-0	18.34564209	-37.9375
magnetic-field-1	-0.410461426	4.125
magnetic-field-2	-44.0032959	-57.5625
gps-0	50.22962952	50.2387632
gps-1	-5.389703751	-5.39141121
photo	files/photo-1428497125-753761.jpg	files/photo-1427720820-588114.jpg

The process of automatically converting the photographs into GIS-ready GeoTIFFs was not straightforward and required several iterations before an acceptable translation was achieved. We evaluated which of the phone’s sensors were most useful for performing the conversion. Initial tests focused on using magnetic field direction sensors but reliance on these alone produced very poor results. Further testing revealed that the phone’s orientation data provided a higher level measurement and generated more useable results because it was the result of ‘sensor fusion’ and combined data from several sensors (typically in a high-end smartphone this would likely include the magnetometer, accelerometer, gyroscope, and compass, but the combination used will vary from model to model). Orientation data were subsequently used to drive the GeoTIFF generation. We tested this approach using the dataset shown in Fig 10, collected from the KAP rig. The highly heterogeneous site (an unused quarry with small ponds, aggregate piles and sand-dune vegetation) contained obvious geographical and landscape features which enabled a visualisation of the quality of the GeoTIFF generation when the geolocated images were displayed in GIS and overlaid for comparison with independent spatial data.

**Fig 13:**
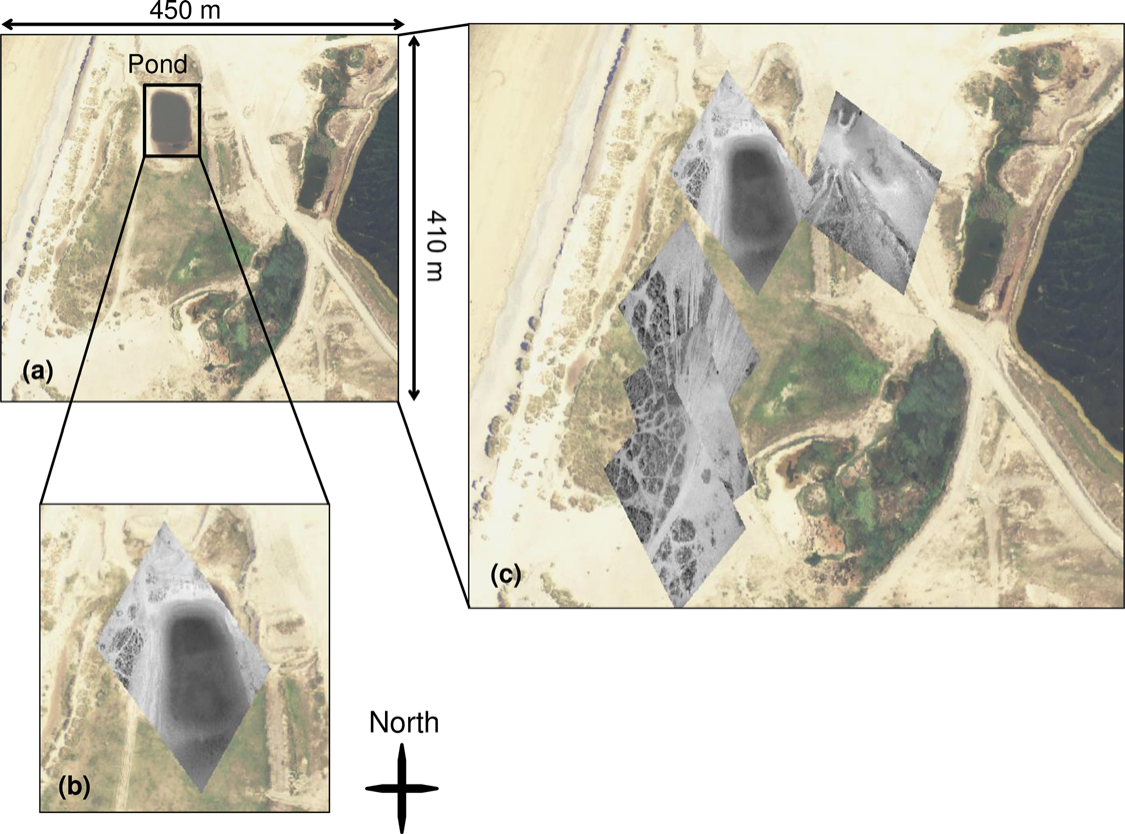
The results of generating GeoTIFF images using the camera photographs coupled with metadata captured simultaneously by the app. (a) Aerial photography dataset (reprinted under a CC BY license with permission from Bluesky International Ltd) image of an unused quarry study site where a KAP platform was flown with the OnePlus One phone; (b) a close-up of a pond showing the GeoTIFF image overlay (in greyscale); (c) further examples of the GeoTIFF positioning for photographs captured at nadir using the app (app images appear as greyscale overlaid on aerial photography dataset (reprinted under a CC BY license with permission from Bluesky international Ltd) in colour.

An example visualisation of a subset of results is shown in Fig 13. A recent aerial photography dataset provided by Bluesky International has been used in Fig 13(A) to show the general area of the KAP survey. A pond (with approximate size 70 m long and 46 m wide) has been highlighted. Fig 13(B) shows one of the GeoTIFF images produced by the workflow described, and using images captured by the app of a freshwater pond (shown in greyscale) overlaid on the arial photography (colour) in QGIS (version 2.10.1 Pisa). Whilst the positioning of the image in Fig 13(B) is not perfect, the image is shown to appear in approximately the right location, and in the correct orientation. The scaling of the pond feature in the GeoTIFF was similar to that measured in the aerial photography dataset but there was a spatial offset of around 14 m in a southerly direction where the top of the pond was displaced too far south in the GeoTIFF. Four further GeoTIFFs have been overlaid on the aerial photography layer in Fig 13(C)–these show the best quality GeoTIFFs generated by our method when the sensors onboard the camera indicated that it was oriented so that the camera was pointing at nadir. The three overlaid images towards the south of the area of interest co-locate very well with each other and appear to show similar patterns in the vegetation cover to the aerial photography layer. The GeoTIFF that is positioned towards the East of the pond is also correctly oriented, positioned and scaled.

The results shown in Fig 13 suggest that the GeoTIFF conversion process holds promise for a seamless integration with GIS. Some issues were encountered with the process–for example, where images were collected off-nadir, the correction was poor because the process could not account for the complex geometry of the platform coupled with the topography of the ground surface. Secondly, the conversion process used here is a first order correction and could be improved by using other sensors to quality filter images or flag potentially erroneous parts of the time-series. For example, where the phone’s sensor set indicates high speeds of movement, or lots of change in attitude over short time scales, there could be an automatic image flag or removal. There is therefore potential for future development and experimentation. Three further limitations of the current method, and probably the causal factors in the positional uncertainty shown in Fig 13 (B and C) are: (a) the poor quality of the phone's GPS position given consumer grade GPS accuracy (c.f. +/-10 m); (b) our need to assume a constant flight height because the elevation records collected by consumer grade GPS receivers have poorer accuracy in z than in x,y; and (c) the impact of unknown angular distortions (caused by the camera lens (barrel distortion) and projection onto the fine scale topography) with the phone images that our algorithm cannot correct. With regards to (b) If the drone or kite’s position is changed by local wind conditions, this will result in biases in both the scaling of the GeoTIFFs and in the positioning of the photograph corners. We postulate that perhaps temporal averaging of the GPS metadata records and hovering over survey points for longer periods of time, would improve the process and sizing of the extent of the images.

## Discussion and Conclusions

This paper has reported the results of a project that has successfully developed a user-friendly and customisable application to allow Android handsets to be converted into a tool for grassroots remote sensing. We have demonstrated typical results that can be delivered by the app from a range of near-range, low cost airborne platforms including fixed wing and multi-rotor drones, stunt and KAP kites. The results show that basic aerial photographs can be captured from all of these platforms but that the highest quality mapping products are achieved when the transference of platform vibrations to the handset are limited.

At this juncture, it is important to return to the intended audience and user base for the app. The app was built to facilitate rapid capture of straightforward remote sensing images from basic platforms, assuming minimal need for specialist engineering. It is important to bear in mind that most scientific users wanting high quality radiometric or spatial data from kite or drone platforms would be unlikely to use a mobile phone as their main imaging device. The target audience for the app is much broader than this–it is intended for use in teaching, or in community led mapping, or in settings where a quick ‘start-up’ for a remote sensing study is needed. For example in humanitarian crisis situations where basic survey data are required to inform emergency relief efforts, it is not necessary to have high quality radiometric data–instead, simple images that inform rescuers or aid workers of the location of stranded people, damaged property or flooded land would be perfectly adequate. Equally, in the aftermath of an earthquake or flood, the app would be very capable of delivering aerial imaging products that would allow the identification of damaged features or impacted areas. When thinking about the app’s potential uses in these situations, it is important to reflect on the field of ‘appropriate technology’ [20]–anyone with an Android handset and a platform to allow that phone handset to get airborne can now use this app to collect geotagged aerial images for basic spatial data capture and mapping. The work presented in this paper provides evidence that the app provides a freely available tool for open source spatial data capture.

Looking at the quality of results generated by the app, we found that the best results were gathered from a KAP rig costing less than £100, where the OnePlus One phone was suspended using a 3D printed plate suspended from the single line of the kite. On motorised platforms the main data quality issue was caused by systematic distortions in the images caused by interference between the imaging sensor and the vibrations of the motor. Both of the mobile phone models tested used the popular CMOS image sensor due to their lightweight, small size and low power consumption [32,36]. One drawback of CMOS sensors is their use of a rolling shutter, whereby a line scanning approach is used to capture an image, meaning that every pixel is not imaged at exactly the same time, increasing the chances of blur due to motion [37]. This risk of blurring is apparent in many of the photographs collected when the platform velocity was high or when high frequency vibrations were present. The results indicate that there is a requirement to provide adequate vibration dampening, flight planning or change in vehicle control to mitigate for this. We have shown that it is possible to successfully dampen the vibrations caused by drone motors during flight using very basic equipment (here, we used a kitchen sponge between the phone and the Quanum Nova multirotor aircraft), but this did not remove all vibrational effects. On a single line kite platform the vibrational effects were not visible in most of the photographs captured, indicating that this offered a more stable platform for image capture with CMOS-camera equipped mobile phones. For high quality mapping from a drone platform, we suggest that the phone would need a more sophisticated vibration-dampened mounting plate. Other grassroots approaches point to a solution where the phone could be hung from the underside of an aircraft or kite using a wooden plate suspended by piano wires for vibration absorption–but doing this requires access to appropriate engineering capabilities and materials, which in some of the situations where this app is intended for use, may not be available. This technique was used by George R. Lawrence for his early aerial photography work from kites [38] but has also been explored in drones as a low cost way of suspending cameras and reducing vibration effects [39,40]. There are changes on the horizon with smartphone manufacturers starting to use different camera sensors within their handsets. For example, Sony have just released a stacked CMOS image sensor with built-in hybrid autofocus and 3-axis electronic image stabilization which may reduce the impacts of platform vibration effects in the future [41].

Critically to its flexible use, the visual coding ‘scheme bricks’ approach used to design and live-code the app will allow end-users to customise their own data acquisition and to a certain extent, to control image quality (e.g. by limiting the conditions under which the camera is triggered to capture a photograph). The live coding capability provides great flexibility for successful deployment on a wide range of Android handsets. We have demonstrated the use of open-source GDAL tools to convert the high quality jpeg images captured by the phone camera into GeoTIFF images for direct use in GIS software, and shown the limitations of the approach as it relies on uncertain GPS positional information and height data.

The recent global expansion of the lightweight consumer drone market, the globally ubiquitous availability of kites and mobile phones, and a growing social appetite for open-source, free to use mapping data means that there is now a great opportunity for this app to be put to great use in democratic and participatory mapping exercises. Anyone in the world with an android handset and access to a vertical space (from a kite, drone or even from a rooftop or terrace looking down) where nadir Earth-focused imaging can be captured can now use this app to generate new fine-grained mapping products. The app can be downloaded freely from the Google store by searching for ‘UAV-toolkit’ [42], and all code is open source and available from [31]. We appreciate any feedback that users provide.
